# Tissue-aware RNA-Seq processing and normalization for heterogeneous and sparse data

**DOI:** 10.1186/s12859-017-1847-x

**Published:** 2017-10-03

**Authors:** Joseph N. Paulson, Cho-Yi Chen, Camila M. Lopes-Ramos, Marieke L. Kuijjer, John Platig, Abhijeet R. Sonawane, Maud Fagny, Kimberly Glass, John Quackenbush

**Affiliations:** 1 Department of Biostatistics and Computational Biology, Dana-Farber Cancer Institute, Boston, MA 02215 USA; 2 Department of Biostatistics, Harvard School of Public Health, Boston, MA 02215 USA; 3 Channing Division of Network Medicine, Brigham and Women’s Hospital and Harvard Medical School, Boston, MA 02215 USA; 4 Department of Cancer Biology, Dana-Farber Cancer Institute, Boston, MA 02215 USA; 5 Present address: Genentech, Department of Biostatistics, Product Development, 1 DNA Way, South San Francisco, CA 94080 USA

**Keywords:** GTEx, RNA-Seq, Quality control, Filtering, Preprocessing, Normalization

## Abstract

**Background:**

Although ultrahigh-throughput RNA-Sequencing has become the dominant technology for genome-wide transcriptional profiling, the vast majority of RNA-Seq studies typically profile only tens of samples, and most analytical pipelines are optimized for these smaller studies. However, projects are generating ever-larger data sets comprising RNA-Seq data from hundreds or thousands of samples, often collected at multiple centers and from diverse tissues. These complex data sets present significant analytical challenges due to batch and tissue effects, but provide the opportunity to revisit the assumptions and methods that we use to preprocess, normalize, and filter RNA-Seq data – critical first steps for any subsequent analysis.

**Results:**

We find that analysis of large RNA-Seq data sets requires both careful quality control and the need to account for sparsity due to the heterogeneity intrinsic in multi-group studies. We developed Yet Another RNA Normalization software pipeline (YARN), that includes quality control and preprocessing, gene filtering, and normalization steps designed to facilitate downstream analysis of large, heterogeneous RNA-Seq data sets and we demonstrate its use with data from the Genotype-Tissue Expression (GTEx) project.

**Conclusions:**

An R package instantiating YARN is available at http://bioconductor.org/packages/yarn.

**Electronic supplementary material:**

The online version of this article (10.1186/s12859-017-1847-x) contains supplementary material, which is available to authorized users.

## Background

RNA-Seq experiments using ultrahigh-throughput sequencing-by-synthesis technologies were first performed in 2008 and have since been used for large-scale transcriptome analysis and transcript discovery in mammalian genomes [1–3]. Although hundreds of published studies have used this technology to assay gene expression, the majority of studies consist of relatively small numbers of samples. There are many widely used methods for normalization and analysis of expression data from modest numbers of relatively homogeneous samples [4–6]. The workflow for RNA-Seq typically includes basic quality control on the raw reads and alignment of those reads to a particular reference database to extract sequence read counts for each feature—gene, exon, or transcript—being assayed [7]. The resulting features-by-samples matrix is then filtered, normalized and analyzed to identify features that are differentially expressed between phenotypes or conditions. Functional enrichment analysis is then performed on these features [7].

There are now many large cohort studies, including the Genotype-Tissue Expression project (GTEx) and The Cancer Genome Atlas (TCGA) that have generated transcriptomic data on large populations and across multiple tissues or conditions to study patterns of gene expression [8, 9]. The GTEx project is collecting genome-wide germline SNP data and gene expression data from an array of different tissues on a large cohort of research subjects. GTEx release version 6.0 sampled over 550 donors with phenotypic information representing 9590 RNA-Seq assays performed on 54 conditions (51 tissues and three derived cell lines). We excluded K562 from our analyses since this leukemia cell line does not represent a healthy tissue and is only a reference cell line unrelated to any GTEx participants. GTEx assayed expression in 30 tissue types, which were further divided into tissue subregions [8]. After removing tissues with very few samples (fewer than 15), we were left with 27 tissue types from 49 subregions. This included 13 different brain regions and three types of skin tissue. While GTEx broadly targeted body regions, the sampling is uneven across these subregions, with some sampled in nearly every donor and others sampled in only a small subset. For example, there are some tissues, such as the brain, in which many subregions were sampled with the expectation that those samples might exhibit very different patterns of expression.

Established methods for RNA-Seq analysis can be used to make direct comparisons of gene expression profiles between phenotypic groups within a tissue. However, they are not well suited for comparisons across multiple, diverse tissues, of which each exhibit a combination of commonly expressed and tissue-specific genes. This characteristic is a feature that confounds most normalization methods, which generally assume the majority of expressed transcripts are common across samples. Widely-used normalization methods make assumptions that are valid only in fairly consistent samples and assume that most genes are not differentially expressed, that housekeeping genes are expressed at equivalent rates, or that the expression distributions vary only slightly due to technology [4–6]. In large heterogeneous data sets, such as GTEx, these biological assumptions are violated. When looking at diverse tissues, or distinct patterns of expression, the use of the appropriate quality control is necessary in order to make valid comparisons of expression profiles.

**Fig. 1:**
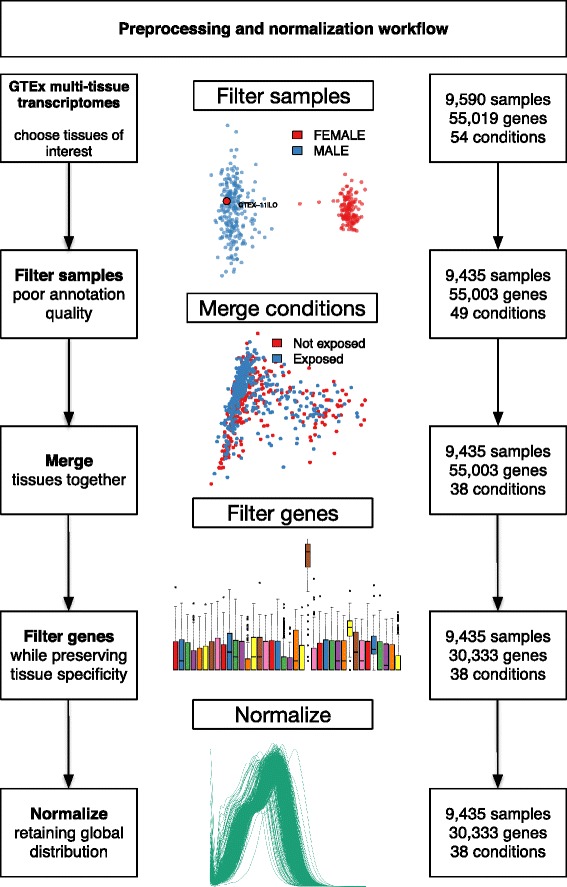
Preprocessing workflow for large, heterogeneous RNA-Seq data sets, as applied to the GTEx data. The boxes on the right show the number of samples, genes, and tissue types at each step. First, samples were filtered using PCoA with Y-chromosome genes to test for correct annotation of the sex of each sample. PCoA was used to group or separate samples derived from related tissue regions. Genes were filtered to select a normalization gene set to preserve robust, tissue-dependent expression. Finally, the data were normalized using a global count distribution method to support cross-tissue comparison while minimizing within-group variability

Yet Another RNA-Seq normalization pipeline (YARN), illustrated in Fig. 1, is a data preprocessing and normalization pipeline that includes filtering poorly annotated samples, merging samples from “states” that have indistinguishable expression profiles, filtering genes in a condition specific manner, and normalizing to keep global distributions while controlling for within group-variability. While every step in the gene-by-sample feature matrix generation process can bias downstream results, our focus in this analysis, and in the YARN package, is on the downstream effects of methods used to filter and normalize data that has already been aligned to a reference genome.

## Implementation

YARN, shown in Fig. 1, is instantiated as a Bioconductor (BioC version 3.4+) R package. YARN is built on top of the Biobase Bioconductor package that defines the ExpressionSet class, a S4 object class structure. Using this class structure, multiple helper functions were designed to help 1) filter poor quality samples – (checkMisAnnotation), 2) merge samples derived from similar sources (in our case, different sampling regions of the “same” tissue) for increased power (checkTissuesToMerge), 3) filter genes while preserving tissue or group specificity – (filterLowGenes, filterGenes, filterMissingGenes), 4) normalize while accounting for global differences in tissue distribution (normalizeTissueAware), and 5) visualize the structure of the data (plotDensity, plotHeatmap, plotCMDS). The full details of our pipeline methodology are available in Additional file 1. The object-oriented architecture allows for future expansion of the pipeline and the ExpressionSet class allows for integration with various other Bioconductor packages. Example data sets have been curated and are available within the packages. The R package instantiating YARN is available at http://bioconductor.org/packages/yarn.

## Results

### Annotation quality assessment

The first step in any good data processing pipeline is quality assessment to assure that samples are correctly labeled. Reliable metadata is critical for studies and a high rate of mis-assignment raises issues about the quality of the rest of the annotation provided for each sample. Some disease states and sex annotation metadata can be checked with the RNA-Seq expression values using disease biomarkers or sex chromosomal genes. Misannotation is a common problem, with 46% of studies potentially having had misidentified samples [10]. We ourselves found it necessary to remove 6% of samples in an analysis of sexual dimorphism in COPD due to potential misannotation of the sex of individual samples [11]. While correct sex assignment is not a guarantee that the rest of the annotation is correct, it provides a testable measure of the quality of sample annotation in a study.

As a measure of the quality of the GTEx annotation, we tested for the fidelity of sample sex assignment. We extracted count values for genes mapped to the Y chromosome in each sample, log_2_-transformed the data, and used Principal Coordinate Analysis (PCoA) with Euclidean distance to cluster individuals within each tissue [12] (Additional file 1). While PCoA is similar to Principal Components Analysis (PCA), PCoA has the advantage that the distance between two samples allows for an intuitive interpretation of the quality and reproducibility of a sample. In addition, any appropriate distance can be substituted and PCoA will preserve distances in the decomposition. In contrast, the correlation-based metric used in PCA cannot identify discrepancies if there are large average shifts in expression.

PCoA clearly separates samples into two groups in every tissue using the Y chromosome genes. However, one subject, GTEX-11ILO, annotated as female, grouped with males in each of the 13 tissue regions for which RNA-Seq data was available (Additional file 2: Figure S1); we excluded GTEX-11ILO from further analysis. We later learned that this individual had undergone sex-reassignment surgery providing evidence that this quality check had appropriately flagged an individual who was genetically male.

The PCA plot in the first step of Fig. 1 and the collected set in Additional file 2: Figure S1 were produced using the functions checkMisAnnotation and plotCMDS in the YARN package. While the majority of variation in the GTEx data was present in the first two components and clearly showed separation between the sexes, as a rule of thumb one should check components until 90% of the variation has been captured in the PCs. The plotCMDS function is structured to return as many components as requested for pairwise scatterplots, and users can adjust the number of PCs to capture the desired level of variation. Helper functions in YARN include filterSamples that can help the user remove specified samples. Examples are included in YARN’s help file and the Bioconductor vignette.

### Merging or splitting sample groups

GTEx sampled 51 body sites (based on morphological definitions) and created two cell lines (fibroblasts from skin and lymphoblastoid cells from whole blood). However, not every site was sampled in every individual. Further, there were often multiple sites sampled from the same “organ” (for example, sun exposed and non-exposed skin, or transverse and sigmoid colon), but the GTEx consortium did not report testing whether such samples exhibited fundamental differences in gene expression or if they were effectively indistinguishable. Our interest in analyzing GTEx was to increase our effective power by maximizing the sample size in each tissue by grouping samples that were otherwise transcriptionally indistinguishable (Fagny et al. 2016, Lopes-Ramos et al. 2016; Sonawane et al. 2017; Chen et al. 2016).

**Fig. 2:**
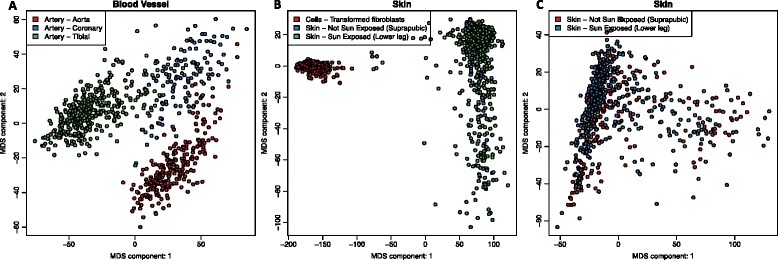
PCoA analysis allows for grouping of subregions for greater power. Scatterplots of the first and second principal coordinates from principal coordinate analysis on major tissue regions. **a** Aorta, coronary artery, and tibial artery form distinct clusters. **b** Skin samples from two regions group together but are distinct from fibroblast cell lines, a result that holds up (**c**) when removing the fibroblasts

We first grouped samples based on GTEx-annotated subregions (labeled SMTS) by taking, for example, all skin-derived samples. We excluded the X, Y, and mitochondrial genes, identified the 1000 most variable autosomal genes, and performed PCoA using Euclidean distance on the log_2_-transformed raw count expression data (see Fig. 2 and Additional file 3: Figure S2). We chose the 1000 most variable genes instead of all genes for computational efficiency; results were relatively insensitive to the absolute number of genes used (Additional file 1).

We then visually inspected the PCoA plots to determine whether subregions were distinguishable from each other based on the two first PCs. If they were, the subregions were considered independent tissues in all downstream analyses (for example, transverse and sigmoid colon were considered distinct). Those regions that could not be resolved were merged to improve the power of downstream analyses. If we observed complex patterns, as described for brain below, we performed multiple rounds of PCoA analysis to assure that we had identified transcriptionally distinct regions. In many cases, we found clear separations between tissue subregions, such as for the various arterial or esophageal subregions, which we retained as separate tissues. However, for other tissues, such as sun-exposed and non-exposed skin, we found no distinguishable difference in the PCoA plots (Fig. 2) and therefore merged these into a single tissue for downstream analysis.

The greatest consolidation occurred in brain, where GTEx had sampled 13 subregions. In examining the PCoA plots, we found that samples from cerebellum and cerebellar hemisphere subregions were indistinguishable from each other, but these were very distinct from the other brain regions. We merged the cerebellum and cerebellar hemisphere subregions (brain cerebellum) and removed these from the remaining brain subregions. We then performed a second PCoA on the remaining regions. We found that basal ganglia (brain basal ganglia) clustered separately from the remaining subregions that did not further separate into other groups (brain other; largely cortex, Additional file 3: Figure S2), leaving three brain regions.

The PCoA clearly separated the fibroblast cell line from skin (Fig. 2b-c and Additional file 3: Figure S2) and the lymphoblastoid cell line from blood (Additional file 3: Figure S2). This result is consistent with previous reports that indicate that cell line generation and growth in culture media produces profound changes in gene expression [13, 14]). A detailed transcriptomic and network analysis of these cell lines and their tissues of origin is provided in [14].

By merging subregions, we increased the effective sample size of several of the tissues allowing downstream analyses, such as eQTL analysis [15] that would not have been otherwise possible. This increase in power was also important in the reconstruction of gene regulatory networks [14, 16–18]. The results of our tissue clustering on the GTEx data are summarized in Table 1.

**Table 1:** Breakdown of tissues, assigned groups, abbreviations used, and sample sizes

Tissue	Abbreviation	Subtissue	Sample size
Adipose subcutaneous	ADS	Adipose - Subcutaneous	380
Adipose visceral	ADV	Adipose - Visceral (Omentum)	234
Adrenal gland	ARG	Adrenal Gland	159
Artery aorta	ATA	Artery - Aorta	247
Artery coronary	ATC	Artery - Coronary	140
Artery tibial	ATT	Artery - Tibial	357
Brain other	BRO	Brain - Amygdala	779
		Brain - Anterior cingulate cortex (BA24)	
		Brain - Cortex	
		Brain - Frontal Cortex (BA9)	
		Brain - Hippocampus	
		Brain - Hypothalamus	
		Brain - Spinal cord (cervical c-1)	
		Brain - Substantia nigra	
Brain cerebellum	BRC	Brain - Cerebellar Hemisphere	254
		Brain - Cerebellum	
Brain basal ganglia	BRB	Brain - Caudate (basal ganglia)	360
		Brain - Nucleus accumbens (basal ganglia)	
		Brain - Putamen (basal ganglia)	
Breast	BST	Breast - Mammary Tissue	217
Lymphoblastoid cell line	LCL	Cells - EBV-transformed lymphocytes	132
Fibroblast cell line	FIB	Cells - Transformed fibroblasts	305
Colon sigmoid	CLS	Colon - Sigmoid	173
Colon transverse	CLT	Colon - Transverse	203
Gastroesophageal junction	GEJ	Esophagus - Gastroesophageal Junction	176
Esophagus mucosa	EMC	Esophagus - Mucosa	330
Esophagus muscularis	EMS	Esophagus - Muscularis	283
Heart atrial appendage	HRA	Heart - Atrial Appendage	217
Heart left ventricle	HRV	Heart - Left Ventricle	267
Kidney cortex	KDN	Kidney Cortex	36
Liver	LVR	Liver	137
Lung	LNG	Lung	360
Minor salivary gland	MSG	Minor Salivary Gland	70
Skeletal muscle	SMU	Muscle - Skeletal	469
Tibial nerve	TNV	Nerve - Tibial	334
Ovary	OVR	Ovary	108
Pancreas	PNC	Pancreas	193
Pituitary	PIT	Pituitary	124
Prostate	PRS	Prostate	119
Skin	SKN	Skin - Not Sun Exposed (Suprapubic)	661
		Skin - Sun Exposed (Lower leg)	
Intestine terminal ileum	ITI	Small Intestine - Terminal Ileum	104
Spleen	SPL	Spleen	118
Stomach	STM	Stomach	204
Testis	TST	Testis	199
Thyroid	THY	Thyroid	355
Uterus	UTR	Uterus	90
Vagina	VGN	Vagina	97
Whole blood	WBL	Whole Blood	444

We used the YARN routines checkTissuesToMerge and plotCMDS functions to generate the PC plots as shown in Fig. 2 and Additional file 3: Figure S2. Similar to checking for misannotation, one can visually inspect the overlap of subregions to determine whether data from similar tissues should be merged or kept separate. We recommend checking multiple components and investigating components up until at least 90% of the variability is explained. Multiple components can be plotted using the plotCMDS function in combination with the R base function, pairs.

### Gene selection and filtering for normalization and testing

Most commonly used normalization methods adjust gene expression levels using a common gene set under the assumption that the general expression distributions are roughly the same across samples. With RNA-Seq experiments, the selection of an appropriate gene set with which to carry out normalization is more challenging because, even when comparing related samples, each sample may have a slightly different subset of expressed genes. Because of this, filtering methods are essential in preprocessing RNA-Seq data to remove noisy measurements and increase power without biasing differential expression results [19].

In the GTEx expression data we found many “tissue-specific” genes that were expressed in only a single or a small number of tissues (Additional file 1, Additional file 4: Figure S3). We tested two different filtering methods: (1): a “tissue-aware” manner in an unsupervised approach recommended by Anders et al. (Anders et al. 2013), and (2) filtering in a “tissue-agnostic” manner to remove genes with less than one count per million (CPM) in half of all samples (Additional file 1).

The tissue-aware method filters genes with less than one CPM in fewer than half of the number of samples of the smallest set of related samples (for GTEx, at least 18 samples since the “smallest” number of samples in any tissue is 36); this leaves 30,333 genes out of the 55,019 mapped transcripts for which reads are available in GTEx. Of these 30,333, 60% (18,328) are classified as protein coding genes and 11% (3220) are pseudogenes. This contrasts with the tissue-agnostic method in which genes are removed if they appear in fewer than half of the total number of samples in the data set; this filtering method retains only 15,480 genes, of which 84% (12,994) are protein coding and 4% (659) are pseudogenes (Additional file 1, Additional file 5: Table S1, Additional file 6: Figure S4).

We tested these filtering strategies and compared the results to unfiltered data by assessing differential expression between whole blood (*n* = 444) and lung (*n* = 360), two tissues with relatively large numbers of samples, and for which we expect to find many differentially expressed genes (Additional file 1). Following filtering, we normalized the data using qsmooth and used voom, from Bioconductor R package limma [20], to identify differentially expressed genes.

We found the smallest fraction of differentially expressed genes in the unfiltered data set (54%). The tissue-agnostic filtering identified the largest fraction (80%), but many of the differentially expressed genes were noncoding genes. The tissue-aware filtered data yielded an intermediate fraction of differentially expressed genes (69%), but the greatest number of differentially expressed protein coding genes. Consequently, we chose to use tissue-aware filtering as it provides for identification of tissue-specific, differentially expressed genes (Additional file 1). Using this filtering with the GTEx data reduced the number of mapped genes from 55,003 to 30,333 genes that were advanced to the next step in the pipeline.

**Fig. 3:**
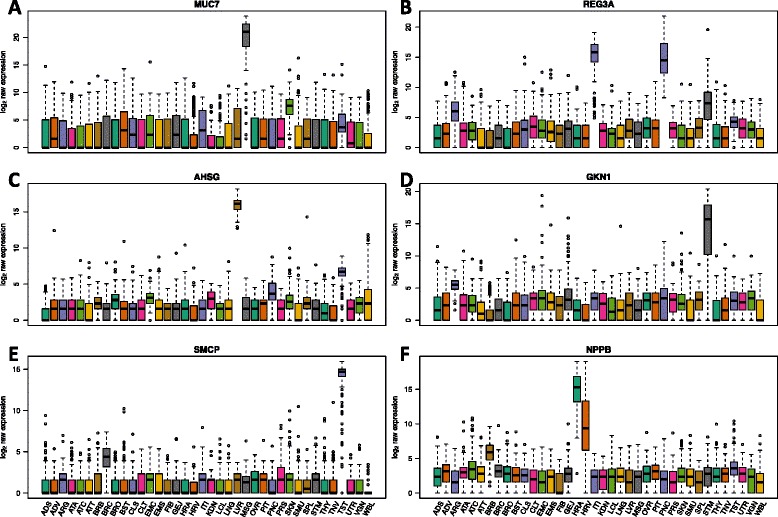
Six highly expressed tissue-specific genes that are removed upon tissue-agnostic filtering. Boxplots of continuity-corrected log_2_ counts for six tissue-specific genes (**a**-**f**). These genes are retained when considering tissue-specificity and not when filtering in an unsupervised manner. Colors represent different tissues. Examples include (**a**) *MUC7,* (**b**) *REG3A*, (**c**) *AHSG*, (**d**) *GKN1*, (**e**) *SMCP*, and (**f**) *NPPB*

Figure 3 shows examples of genes related to tissue-specific function or disease that would have been lost using the tissue-agnostic approach that are retained by the tissue-aware filtering. *MUC7* (Fig. 3a) is overexpressed in the minor salivary gland and has been associated with asthma. *REG3A* (Fig. 3b) is overexpressed in pancreas and small intestine and has been associated with cystic fibrosis and pancreatitis. *AHSG* (Fig. 3c) is overexpressed in the liver and has been associated with uremia and liver cirrhosis. *GKN1* (Fig. 3d) is overexpressed in the stomach and is downregulated in gastric cancer tissue as compared to normal gastric mucosa. *SMCP* (Fig. 3e) is overexpressed in the testis, where it is involved in sperm motility. It is also linked to infertility and tumorigenicity of cancer stem-cell populations [21, 22]. *NPPB* (Fig. 3f) is overexpressed in the heart left ventricle and heart atrial appendage and has been associated with systolic heart failure. Retaining such tissue-specific genes is crucial for understanding the relationship between gene expression and tissue-level phenotypes and understanding their impact on the complex biological system [17].

In YARN, multiple functions are available for filtering lowly expressed genes, including, filterLowGenes, filterMissingGenes, and filterGenes. These functions allow for filtering genes by either a minimum CPM threshold (tissue-aware/agnostic approach), those that are missing, or those mapping to a specific chromosome, respectively. The use of these functions helps retain tissue-specific genes while removing extremely low abundance genes that may represent sequencing noise [19, 23] (Additional file 1).

### Tissue-aware normalization

Normalization is one of the most critical steps in data preprocessing and there are many normalization approaches that have been used in expression data analysis. Many early and widely used methods for RNA-Seq normalization were based on scaling [24–26]. More recently developed methods such as voom [20] use quantile normalization, which assumes that all samples should express nearly identical sets of genes with similar distributions of expression levels. Although quantile normalization has proven to be a robust approach in many microarray applications, its assumptions break down when analyzing samples in which gene expression can be expected to be substantially different among members.

Quantile normalization forces every sample’s statistical distribution to the reference’s distribution where the reference is defined as the average of all sample count quantiles. When the distributional shapes are dissimilar across tissues, the reference is not representative of any particular tissue and scaling of quantiles is dependent on the largest tissue’s distribution. In GTEx, we wanted to use a single normalization method for all tissues. Here, with a very diverse set of tissues, the assumptions underlying quantile normalization clearly break down (Additional file 4: Figure S3).

The qsmooth [27] normalization method is a generalization of quantile normalization that normalizes all samples together but relaxes the assumption that the statistical count distribution should be similar across all samples and instead assumes only that it is similar in each phenotypic group (as one might expect for different tissues in GTEx). We used qsmooth to normalize the GTEx expression data where phenotypic groups were determined using the 38 “merged” tissues that resulted from our quality control assessment.

**Fig. 4:**
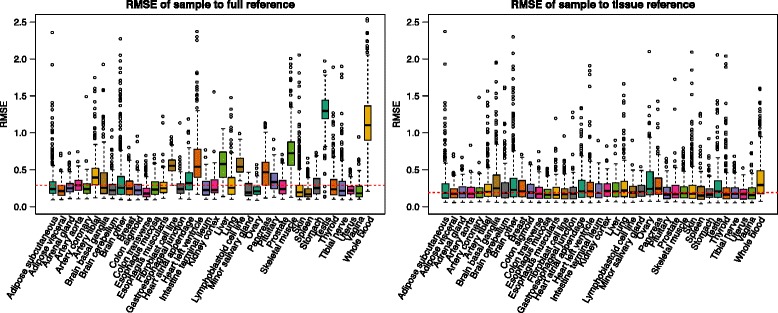
Using a tissue-defined reference lowers root mean squared error. Boxplots of the RMSE comparing the log-transformed quantiles of each sample to the reference defined using (left) all tissues and samples and the (right) reference defined using samples of the same tissue

We compared the effects of “full” quantile normalization to the “tissue-specific” strategy implemented in qsmooth. We observed much larger root mean squared errors (RMSE) using an all-sample reference (“full” quantile normalization) than we saw using qsmooth’s tissue-specific references (Fig. 4). The root mean square error estimates the divergence of transcriptome distributions from the assumed transcriptome reference distribution. The more the RMSE varies by tissue, the larger the number of tissue-specific counts. Figure 4 suggests that global quantile normalization disproportionately weights and biases tissue-specific transcripts based on other tissues’ proportion of zeros in the distribution and tissue sample size (Additional file 1, Additional file 7: Figure S5). Both qsmooth (smooth quantile normalization) and full quantile normalization (over every specific tissue) are implemented in YARN’s normalizeTissueAware function.

## Discussion

Large-scale transcriptional studies, such as GTEx, present unique opportunities to compare expression in a relatively large population and across a large number of tissues. However, as with all analyses of gene expression, it requires careful quality assessment, gene filtering, and normalization if meaningful conclusions are to be drawn from the data. We developed a simple and robust software pipeline, YARN, to allow us to perform quality control assessment of the metadata associated with a large, heterogeneous data sets such as the collection of RNA-Seq assays that are available as part of the GTEx v6 release.

YARN was designed to process RNA-Seq data to allow comparisons between diverse conditions and consists of four basic steps: quality assessment filtering to remove questionable samples, comparison of “related” sample groups to merge them or split them into separate groups, filtering genes that have too few counts while preserving tissue-specific genes, and normalizing the data. For each step, YARN contains multiple options that allow user to adapt the pipeline for their use.

In our analysis of GTEx v6 data, we began by using PCoA to filter samples based on misidentification by sex. We then used PCoA to compare samples from the same general body site so as to merge those that were indistinguishable. Next, we used a tissue-aware filtering method to retain genes that were expressed in one or a small number of tissues, while eliminating those in too few samples to perform a reliable normalization. Finally, we used qsmooth to perform a tissue-aware normalization (Additional file 1).

This pipeline allowed us to identify one individual who was misidentified by sex, to reduce the 53 sampling site conditions to 38 non-overlapping tissues, eliminated 24,670 genes for which there was insufficient data to perform a reasonable normalization or subsequent analysis, and to produce normalized data for 30,333 genes in 9435 samples distributed across 38 tissues. The result of applying YARN is a data set in which general expression levels are comparable between tissues, while still preserving information regarding the tissue-specific expression of genes. This comparability allowed us to use the normalized data in a wide range of analyses that compared processes across tissues [14, 15, 17, 18].

## Conclusions

YARN is a flexible software pipeline designed to address a problem that is becoming increasingly challenging—that of normalizing increasingly large, complex, heterogeneous data sets, often consisting of many samples representing many different physical states, perturbations, or phenotype groups. YARN is implemented as a Bioconductor package and is available under the open source GPL v3 license at http://www.bioconductor.org/packages/yarn.

The workflow includes numerous quantitative options for filtering as well as tools for visual inspection of data to allow users to understand the distributional and other characteristics of the data. The Bioconductor vignette includes sample skin data from GTEx that can be used to work through as an example analysis. Example code to reproduce the figures in this manuscript is available through GitHub at: https://github.com/QuackenbushLab/normFigures *.* We intend to actively maintain YARN, adding additional features and integrating it with differential gene expression and analysis tools in Bioconductor.

## Availability and requirements

Project name: Yet Another RNA Normalization software pipeline (YARN).

Project home page: http://bioconductor.org/packages/yarn

Operating system(s): Platform independent.

Programming language: R.

Other requirements: Dependencies: Biobase. Imports: biomaRt, downloader, edgeR, gplots, graphics, limma, matrixStats, preprocessCore, readr, RColorBrewer, stats, quantro. Suggests: knitr, rmarkdown, testthat (> = 0.8).

License: GPLv3.

Any restrictions to use by non-academics: None.

## Additional files

Additional file 1: Supplementary Material for Tissue-aware RNA-Seq processing and normalization for heterogeneous and sparse data. (DOCX 37 kb)

Additional file 2: Figure S1. PCoA analysis of multiple tissues on Y-chromosomal genes can highlight poor sex annotation, related to Fig. 1 and misannotation section. Scatterplots of the first and second principal components from principal component analysis on all major tissue regions. We plotted data from 13 tissue regions from the GTEx consortium, coloring the annotated sex of each sample. Enlarged is sample GTEX-11ILO that clusters with male samples in every tissue despite being annotated as being from a female; we later learned that this research subject was genetically male. (PDF 240 kb)

Additional file 3: Figure S2. PCoA analysis of multiple tissue groups, related to Figs. 1, 2 and merging conditions section. Scatterplots of the first and second principal components from principal component analysis on all major tissue groups colored by sampled region. The grouping in these plots led us to either merge regions into a single group or to keep them separate. The final tissue set used for further analysis is summarized in Table 1. (PDF 73 kb)

Additional file 4: Figure S3. Animated density plots of log-transformed counts when including more tissues, related to Fig. 1. GIF animation of density plots when including 10 largest sample size tissues. As more samples are included we observe a larger fraction of tissue-specific genes as can be seen by the growing spike-in the distribution at zero within each tissue. (GIF 3641 kb)

Additional file 5: Table S1. Breakdown of gene types remaining in each data set after different filtering approaches. Filtering in a tissue-specific manner, we keep genes that appear in a least half the number of samples present in of the smallest phenotype group (for GTEx, at least 18 samples since the “smallest” tissue has 36 total samples); this leaves 30,333 genes of which 60% (18,328) are classified as protein coding genes and 11% (3220) are pseudogenes. This contrasts with our tissue-agnostic method in which genes are removed if they appear in fewer than half of the samples in the data set; this retains only 15,480 genes for which 84% (12,994) are protein coding, and 4% (659) are pseudogenes. (XLSX 36 kb)

Additional file 6: Figure S4. Heatmap of the 15 most variable genes in the GTEx heart samples post filtering, related to Figs. 1 and 3. Heatmap of the 15 most variable genes in the GTEx heart samples. Left, top 15 genes were chosen in an unsupervised manner using the normalized gene expression after a stringent filtering in a tissue-agnostic manner. Right, the 15 most variable genes were chosen in an unsupervised manner using the normalized gene expression after tissue-specific filtering. (PDF 277 kb)

Additional file 7: Figure S5. Count distributions pre- and post- normalization, related to Figs. 1 and 4. Density plots of gene count distributions. Left to right: log_2_ raw expression distribution of samples pre-normalization; count distribution for each sample normalized in a tissue-aware manner. Colors represent different tissues. (PDF 7035 kb)

## Abbreviations

CPM: Count per million
eQTL: Expression quantitative trait loci
GTEx: Genotype-Tissue Expression project
PCA: Principal Components Analysis
PCoA: Principal Coordinate Analysis
PCs: Principal components
RMSE: Root mean squared error
TCGA: The Cancer Genome Atlas
YARN: Yet Another RNA-seq program
