# Supplementary material for: Tissue-aware RNA-Seq processing and normalization for heterogeneous and sparse data
Source: BMC Bioinformatics. 2017 Oct 3;18:437. doi: 10.1186/s12859-017-1847-x (PMC5627434; doi:10.1186/s12859-017-1847-x)
Supplement: Supplementary file 1 — Supplementary Material for Tissue-aware RNA-Seq processing and normalization for heterogeneous and sparse data. (DOCX 37 kb) [file 12859_2017_1847_MOESM1_ESM.docx]

**Supplementary Material for Tissue-aware RNA-Seq processing and normalization for heterogeneous and sparse data**

Joseph N. Paulson^1,2,†^, Cho-Yi Chen^1,2^, Camila M. Lopes-Ramos^1,2^, Marieke L. Kuijjer^1,2^, John
Platig^1,2^, Abhijeet R. Sonawane^3^, Maud Fagny^1,2^, Kimberly Glass^1,2,3^, John Quackenbush^1,2,3,4*^

^1^Department of Biostatistics and Computational Biology, Dana-Farber Cancer Institute, Boston, MA 02215, USA

^2^Department of Biostatistics, Harvard School of Public Health, Boston, MA 02215, USA

^3^Channing Division of Network Medicine, Brigham and Women’s Hospital and Harvard Medical School, Boston, MA 02215, USA

^4^Department of Cancer Biology, Dana-Farber Cancer Institute, Boston, MA 02215, USA

^†^Present address: Genentech, Product Development Biostatistics, 1 DNA Way, South San Francisco, CA 94080, USA

**Supplementary Methods**

**Obtaining data**

We downloaded the Genotype-Tissue Expression (GTEx) version 6.0 RNA-Seq data set (phs000424.v6.p1, 2015-10-05 released) from dbGaP (approved protocol #9112). Excluding the K562 leukemia cell line, GTEx release version 6.0 sampled over 500 donors with phenotypic information and included 9,590 RNA-Seq assays. GTEx assayed expression in 30 tissue types, which were further divided into 53 tissue subregions (51 tissues and two derived cell lines). After removing tissues with fewer than 15 samples, we were left with 27 tissue types from 49 subregions.

According to the GTEx portal, RNA-Seq was performed using the Illumina TruSeq library construction protocol, which is a non-strand specific polyA+ selected library. The sequencing produced 76-bp paired end reads. Following sequencing, alignment to the HG19 human genome was performed using Tophat v1.4.1 assisted by the GENCODE v19 transcriptome definition. It should be noted that the alignment process introduces its own biases. However, we did not revisit the alignment methodology as our interest was in optimizing downstream processing. YARN assumes one already has generated a gene-by-sample count matrix that will be used in subsequent analyses.

YARN includes a function called downloadGTEx to download the publicly available RNA-Seq expression data from the GTEx portal (https://www.gtexportal.org). It should be noted that there are differences between the GTEx data available through the GTEx portal and what is available from dbGaP so the analysis presented here will not be fully reproducible without access to the primary GTEX data at dbGaP.

**Quality control / Filtering samples**

Before normalization, we checked each body site subregion separately for sample misannotation using the YARN functions checkMisAnnotation and plotCMDS. We extracted count values for genes mapped to the Y chromosome in each sample and log_2_-transformed the data and used Principal Coordinate Analysis (PCoA) with Euclidean distance [1]. We visually inspected the first two PCs in scatterplots to identify misannotation based on sex. In GTEx, only a single sample, GTEx-IL10, was identified as misannotated and removed from analysis.

**Merging sample groups**

The GTEx consortium sampled multiple sites from related subregions without checking whether two subregions from the same tissue or organ were distinguishable. This resulted in an artificial splitting of the data into more phenotype groups than necessary, diminishing power for downstream analysis.

We examined each major tissue region separately, defining “tissues” based on the GTEx sample annotation provided by dbGaP in the “SMTS” field. We grouped samples from the “same” tissue, and in each tissue group we identified the 1,000 most variable genes. We used YARN’s checkTissuesToMerge and plotCMDS to perform PCoA and used the PCs to assess how similar samples from subregions of the same “tissue” were to each other. We merged those tissue subregions where the PC plots were inseparable from each other. For tissues such as brain, which had many regions, we re-examined groups after separation to search for additional structure. We recommend analyzing multiple PCs if no clear separation is observed in the first two PCs and those PCs represent less than 90% of the variability.

**Filtering lowly expressed genes**

Genes that are expressed in only a small subset of samples from a single tissue present challenges for any normalization scheme. A commonly used strategy to address this situation is to filter genes that are expressed in a small minority of samples. But there is no clear consensus on how to set the threshold. We compared two filtering strategies, tissue-aware and tissue-agnostic, to unfiltered data.

The unfiltered data included all genes for which expression information was available in any sample; we used the filterMissingGenes function in YARN and collected the data into a genes-by-samples matrix. For a matrix of *g* genes and *N* samples, the “tissue-agnostic” filtering strategy removes genes with fewer than one count per million (CPM) in half of all samples. CPM is calculated as the proportion of an individual gene *i*’s expression, *x_i_*, plus an offset $\epsilon$ (usually 0.25), divided by the sum of genes within the sample $\sum_{i}^{g} x_{i}+\epsilon$, multiplied by 1,000,000.

$$cpm_{i}=\frac{x_{i}+\epsilon}{\sum_{i}^{n} x_{i}+\epsilon}*1000000$$

We used the cpm function from Bioconductor’s edgeR package to calculate the CPM matrix. As a result, the final tissue-agnostic strategy retains a given gene *i*, if $\sum_{1}^{n} I\left( cpm_{i}>1 \right)\sum_{1}^{n} I\left( cpm_{i}>1 \right)\geq\lceil N/2\rceil$.$\left\lceil N/2 \right\rceil$ For example, in a study of 1000 samples, only genes with at least 500 samples having a CPM greater than 1 were kept.

The “tissue-aware” filtering is similar to the “tissue-agnostic” method, but instead of requiring a gene to have a non-zero expression level in half the total number of samples, the tissue-aware method requires that we observe it in at least half as many samples as the smallest tissue or group. In the GTEx expression dataset, the smallest number of samples was in Kidney Cortex, which had 36 samples; therefore, we kept all genes that appeared in at least 18 samples.

**Normalizing in a tissue-aware manner**

We used our tissue group assignments from the PCoA analysis and the genes that passed the tissue-aware normalization as input to the normalization step in YARN. The normalizeTissueAware function in YARN is a wrapper for both the qsmooth function (from the qsmooth package; available on Github at https://github.com/stephaniehicks/qsmooth) and the normalize.quantiles function (in Bioconductor’s preprocessCore package). When comparing the RMSE against the references, we defined references according to the full data set or each tissue as described in the quantile normalization algorithm, the average of sample quantiles [2].

##### Supplementary material for determining the optimal gene set for normalization

One test of how well normalization methods perform is how well one can use downstream analysis methods can use the normalized data to develop meaningful biological insight into the systems being studied. Here we present a brief survey of sex-specific and tissue-specific gene discovery.

##### Impact of filtering on the characterization of the gene types in GTEx data

We began our investigation by analyzing the gene types retained after each filtering. Ideally, the majority of genes filtered are abundant and protein coding. The full GTEx data set provided counts for a total of 55,019 distinct genes based on alignment to the human reference genome GRCh37.p13. Of those, 36% (19,551) were classified as protein coding and 25% (13,666) were classified as pseudogenes, with the remaining 39% classified as antisense, long non-coding RNA (lncRNAs), and others. Filtering in a tissue-aware manner preserved 94% of the protein coding genes compared to 66% when filtering agnostically. Additionally, the ratio of protein coding genes to pseudogenes is 5.4 compared to 1.2, Table S1. Many of the genes filtered in the third approach are tissue-specific, including, *MUC7*, *SMCP*, *REG1A*, *REG3A*, *GKN1*, and *NPPB*, which are abundant in particular tissues and associated with numerous diseases, including cancer, pancreatitis, systolic heart failure, liver cirrhosis, asthma, and others. We highlight a few of these genes in Figure 3. Given many genes that are tissue-specific happen to be filtered we investigated the impact on subregions in the GTEx data.

##### Impact of filtering on differentiation of subregions

Filtering that is too stringent can remove the genes that best differentiate subregions. We calculated and plotted the 25 most variable genes in tissues from the same major sampling regions after both tissue-agnostic and tissue-aware filtering. We observed that many genes distinguishing subtypes are lost when filtering in a tissue-agnostic manner. For example, stringently filtering genes resulted in the loss of many of the most variable genes in arterial samples, namely homeobox genes that differentiated the aorta, tibial, and coronary. Homeobox genes are well characterized and important for cardiovascular development [3]. We filtered in a tissue-specific manner and observed a clear separation of sub-tissue types whereas we did not when filtering in a tissue-agnostic manner (Figure S4).

##### Impact of filtering on sparsity and the relationship between gene presence and library size

Many high-throughput assays have displayed relationships between sequencing depth and the number of observed features, including in metagenomics and single-cell sequencing [4, 5]. Similar to other large sequencing assays, we observed a positive relationship between the number of detected genes and sequencing depth in the GTEx data. This linear relationship between sequencing depth and the number of observed genes is diminished when performing tissue-agnostic filtering (Pearson correlation = 0.09) compared to a tissue-aware filtering (Pearson correlation = 0.25) when removing tissue-specific genes and retaining solely ubiquitous housekeeping genes. The relationship is not surprising given the observation that there is a large increase in the proportion of low or zero valued counts when iteratively including multiple tissues and plotting the count distribution densities (Figure S3). The background set of genes varies across tissues and as a consequence each tissue has varying levels of sparsity. The raw expression profiles and tissue-aware filtering datasets had higher levels of sparsity (raw - 49% average, tissue-aware - 19% average). In contrast, the tissue-agnostic filtering retained non-sparse matrices of less than 1% sparsity on average. Each tissue’s sparsity ranged as well, for example, testis sparsity levels average around 4% while whole blood sparsity levels average at 29%. As a consequence, models that account for sparsity due to either technical variability or tissue-specificity can be beneficial with large RNA-Seq datasets.

##### Impact of filtering on number of differential expressed genes

To further investigate the effect of filtering, we performed differential expression analysis following smooth quantile normalization on two different tissues at each of our filtering levels: no filtering, tissue agnostic, and tissue-specific aware filtering. We compared two of the larger tissues, whole blood (n=444) and lung (n=360). With fewer genes to test there are fewer tests to account, allowing for greater leniency in significance. As a consequence, we observed fewer genes differentially expressed in the unfiltered dataset (54%), compared to the tissue-aware filtered dataset (69%), and the tissue-agnostic filtering (80%).

We obtained the greatest number of differentially expressed protein coding genes when filtering in a tissue-aware manner. This is despite the increased proportion of genes considered differentially expressed in the most stringently filtered dataset and the greater number of potential candidates of the unfiltered dataset. We observed 12,865, 13,397, and 10,427 protein coding genes when unfiltered, tissue-aware, and tissue-agnostic filtering, respectively. The tissue-aware filtering sweet spot allows for a greater number of significant differentially expressed gene calls, both due to the smaller number of tests to correct for and due to an increased pool of tissue-specific genes.

References:

1. Gower JC: **Some Distance Properties of Latent Root and Vector Methods Used in Multivariate Analysis**. *Biometrika* 1966, **53**:325–338.

2. Bolstad BM, Irizarry RA, Astrand M, Speed TP: **A comparison of normalization methods for high density oligonucleotide array data based on variance and bias**. *Bioinformatics* 2003, **19**:185–193.

3. Patterson KD, Cleaver O, Gerber W V., Grow MW, Newman CS, Krieg PA: **1 Homeobox Genes in Cardiovascular Development**. In *Current Topics in Developmental Biology*. *Volume 40*; 1998:1–44.

4. Paulson JN, Stine OC, Bravo HC, Pop M: **Differential abundance analysis for microbial marker-gene surveys**. *Nat Methods* 2013, **10**:1200–1202.

5. Hicks SC, Teng M, Irizarry RA: *On the Widespread and Critical Impact of Systematic Bias and Batch Effects in Single-Cell RNA-Seq Data*. 2015.
