# Supplementary figures and images for: Tissue-aware RNA-Seq processing and normalization for heterogeneous and sparse data
Source: BMC Bioinformatics. 2017 Oct 3;18:437. doi: 10.1186/s12859-017-1847-x (PMC5627434; doi:10.1186/s12859-017-1847-x)

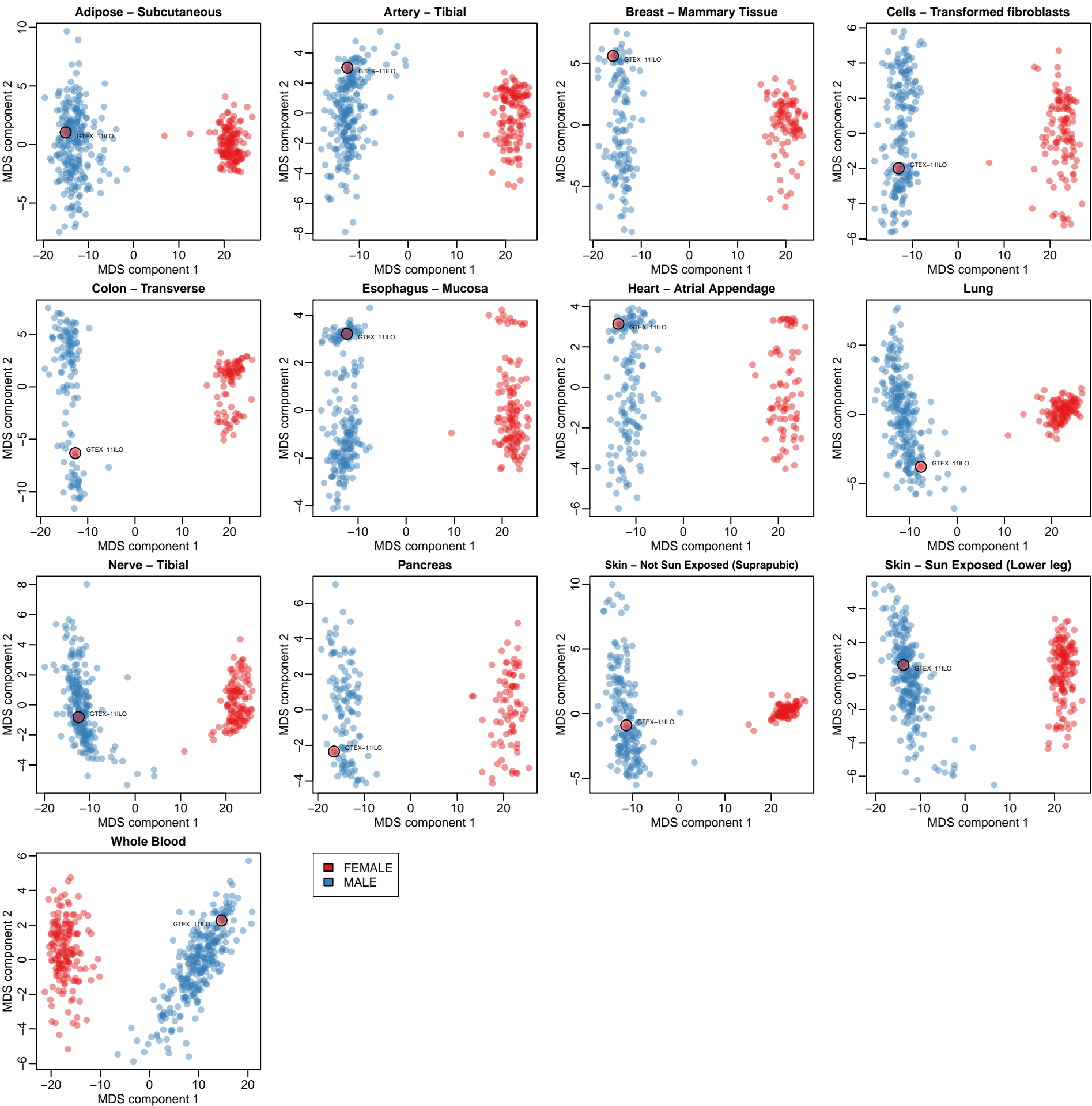

Supplement: Supplementary file 2 — PCoA analysis of multiple tissues on Y-chromosomal genes can highlight poor sex annotation, related to Fig. 1 and misannotation section. Scatterplots of the first and second principal components from principal component analysis on all major tissue regions. We plotted data from 13 tissue regions from the GTEx consortium, coloring the annotated sex of each sample. Enlarged is sample GTEX-11ILO that clusters with male samples in every tissue despite being annotated as being from a female; we later learned that this research subject was genetically male. (PDF 240 kb) [file 12859_2017_1847_MOESM2_ESM.pdf]

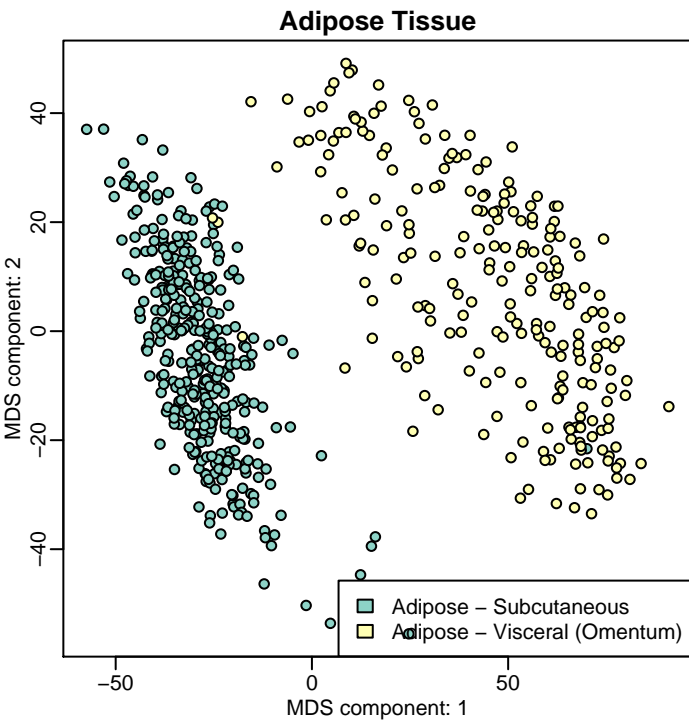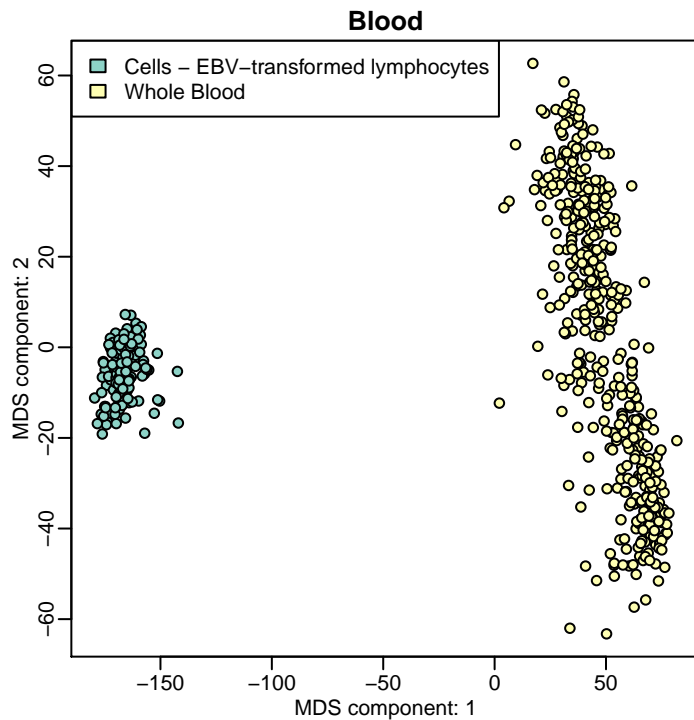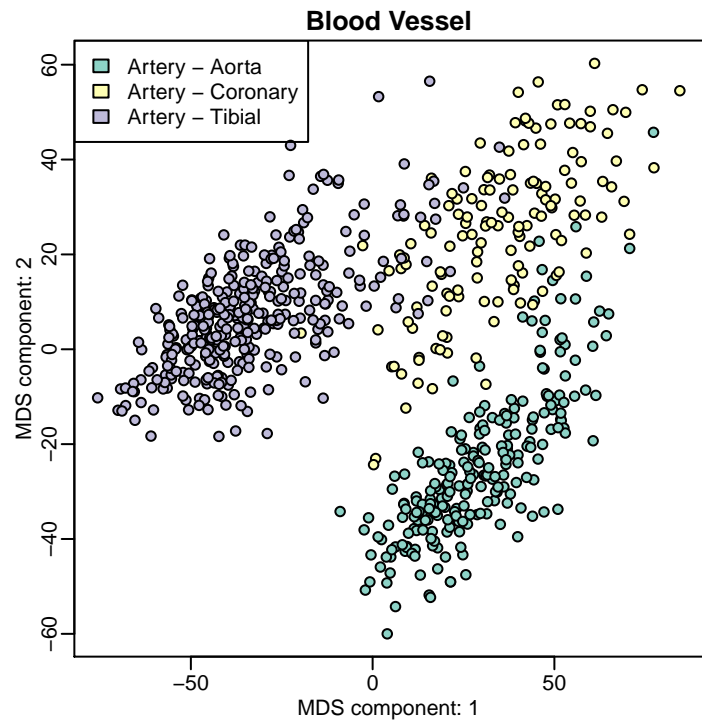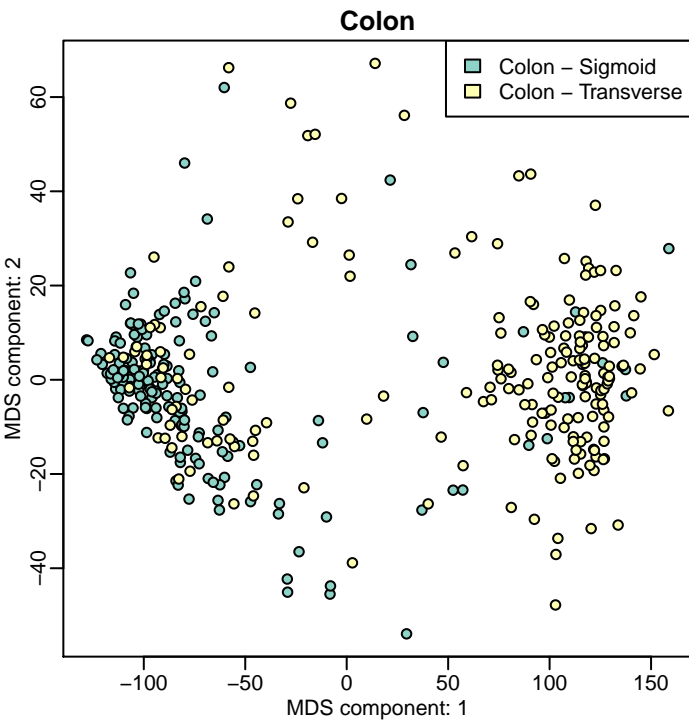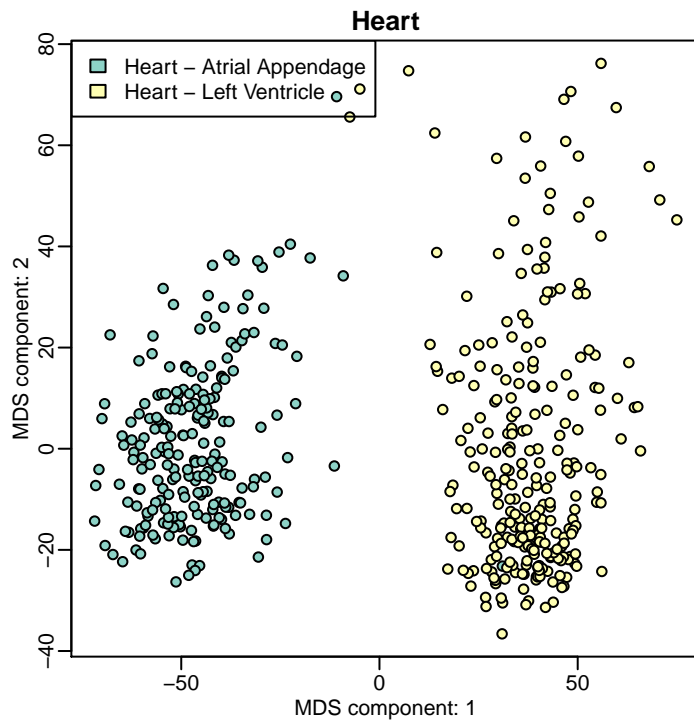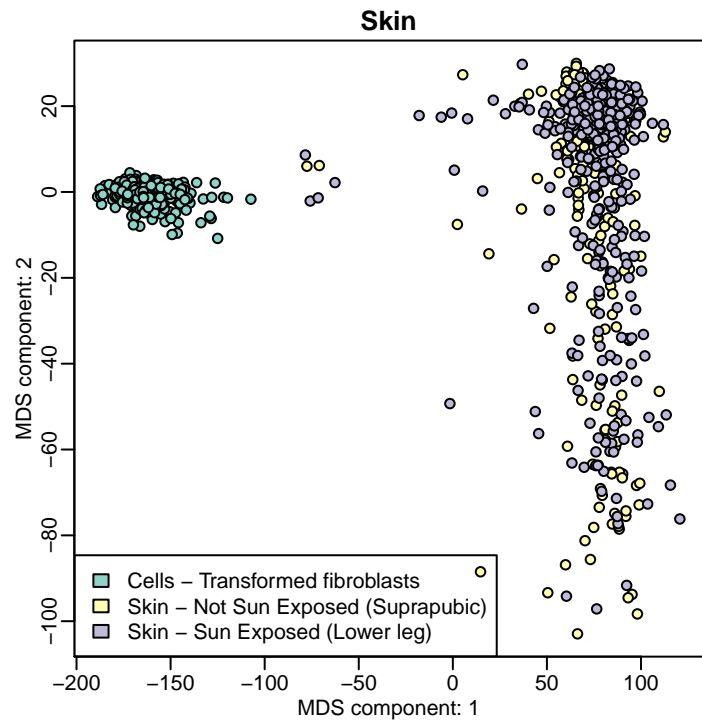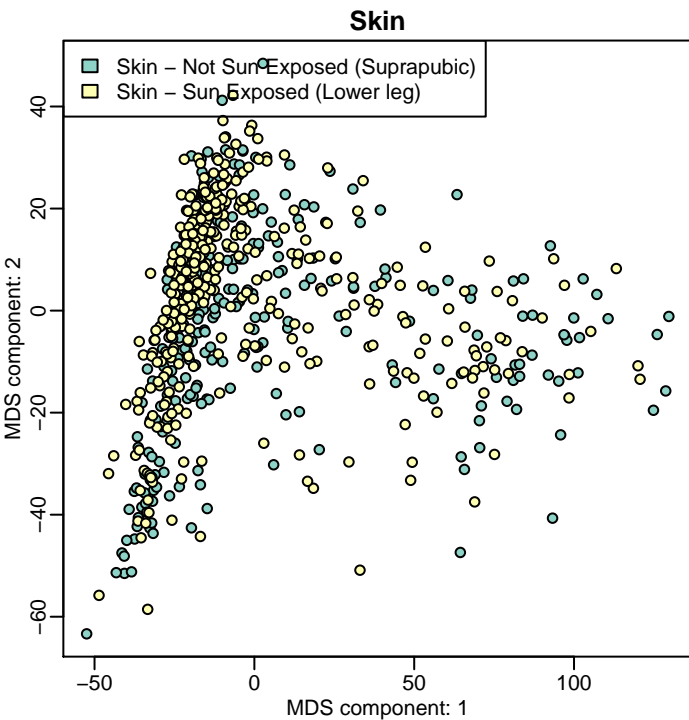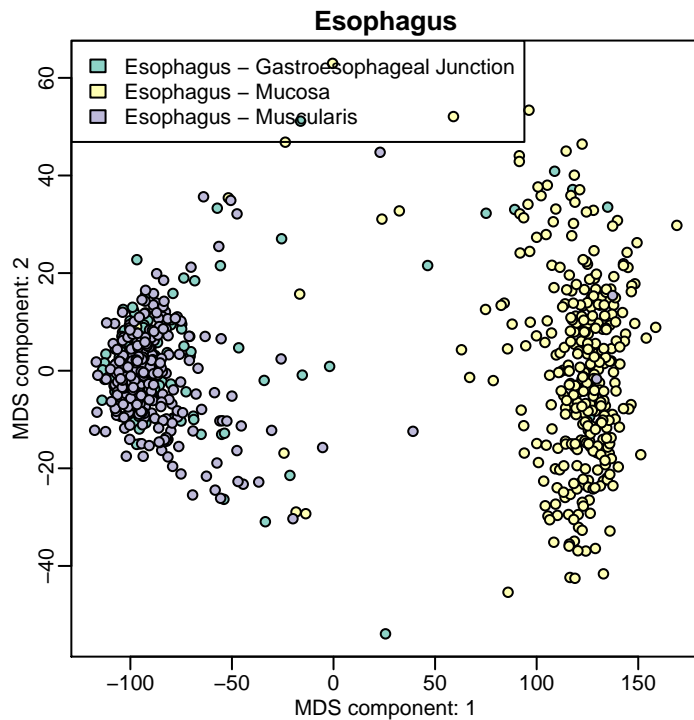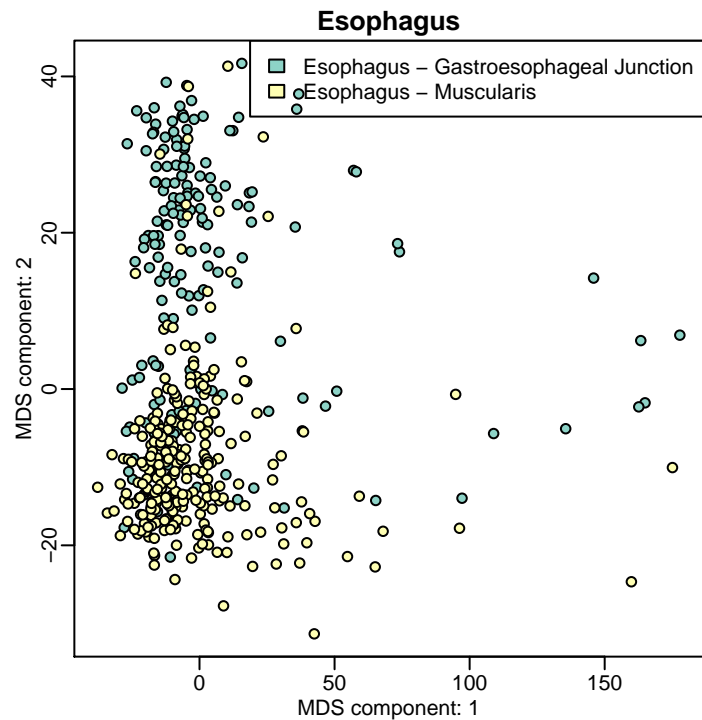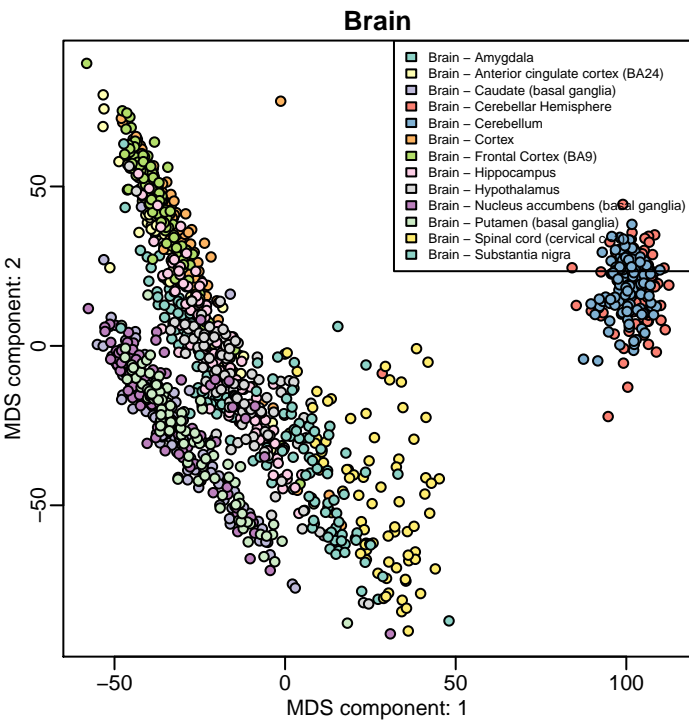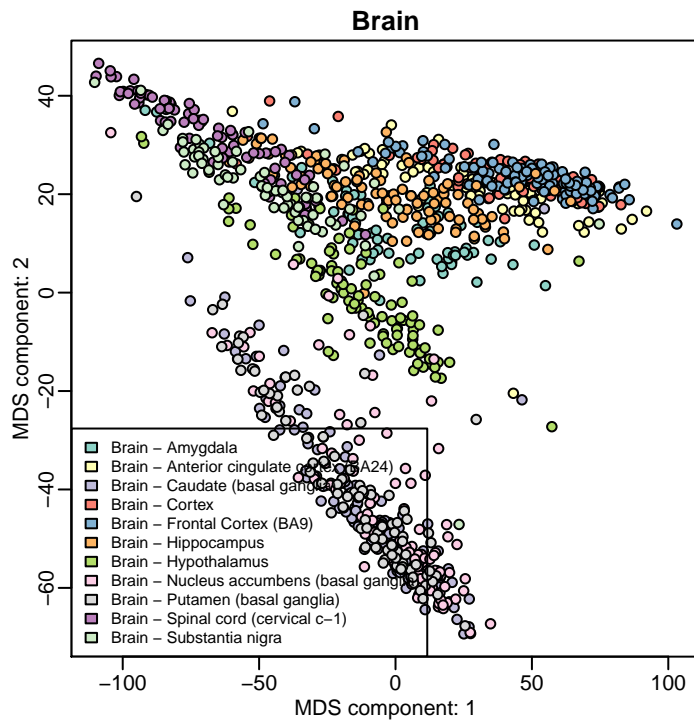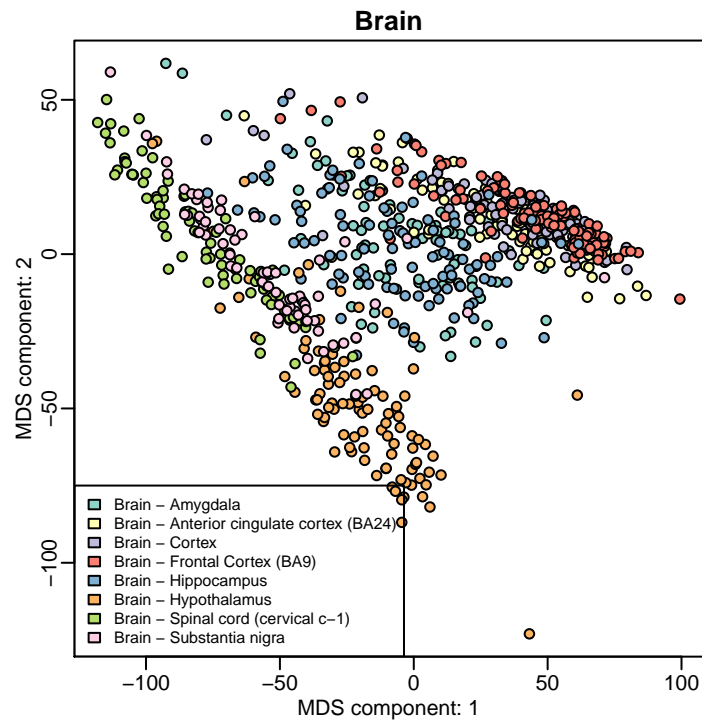

Supplement: Supplementary file 3 — PCoA analysis of multiple tissue groups, related to Figs. 1, 2 and merging conditions section. Scatterplots of the first and second principal components from principal component analysis on all major tissue groups colored by sampled region. The grouping in these plots led us to either merge regions into a single group or to keep them separate. The final tissue set used for further analysis is summarized in Table 1. (PDF 73 kb) [file 12859_2017_1847_MOESM3_ESM.pdf]

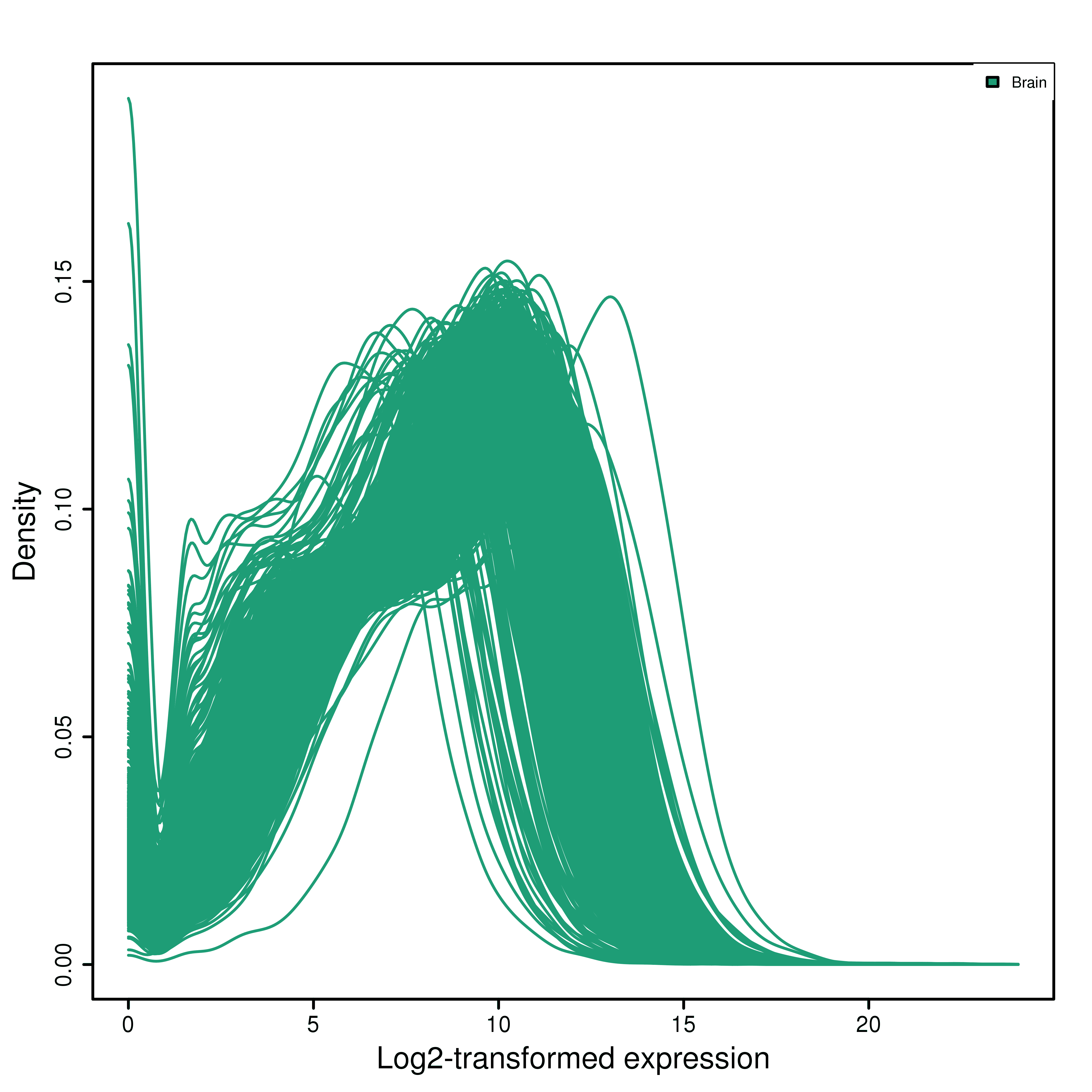

Supplement: Supplementary file 4 — Animated density plots of log-transformed counts when including more tissues, related to Fig. 1. GIF animation of density plots when including 10 largest sample size tissues. As more samples are included we observe a larger fraction of tissue-specific genes as can be seen by the growing spike-in the distribution at zero within each tissue. (GIF 3641 kb) [file 12859_2017_1847_MOESM4_ESM.gif]

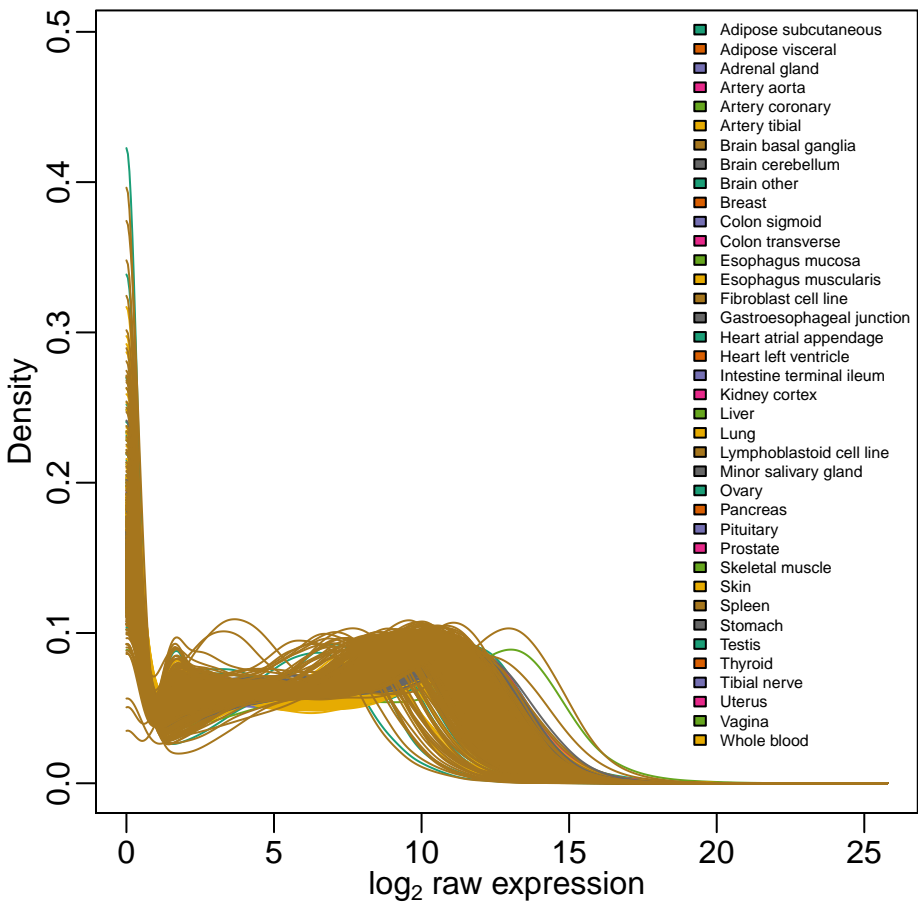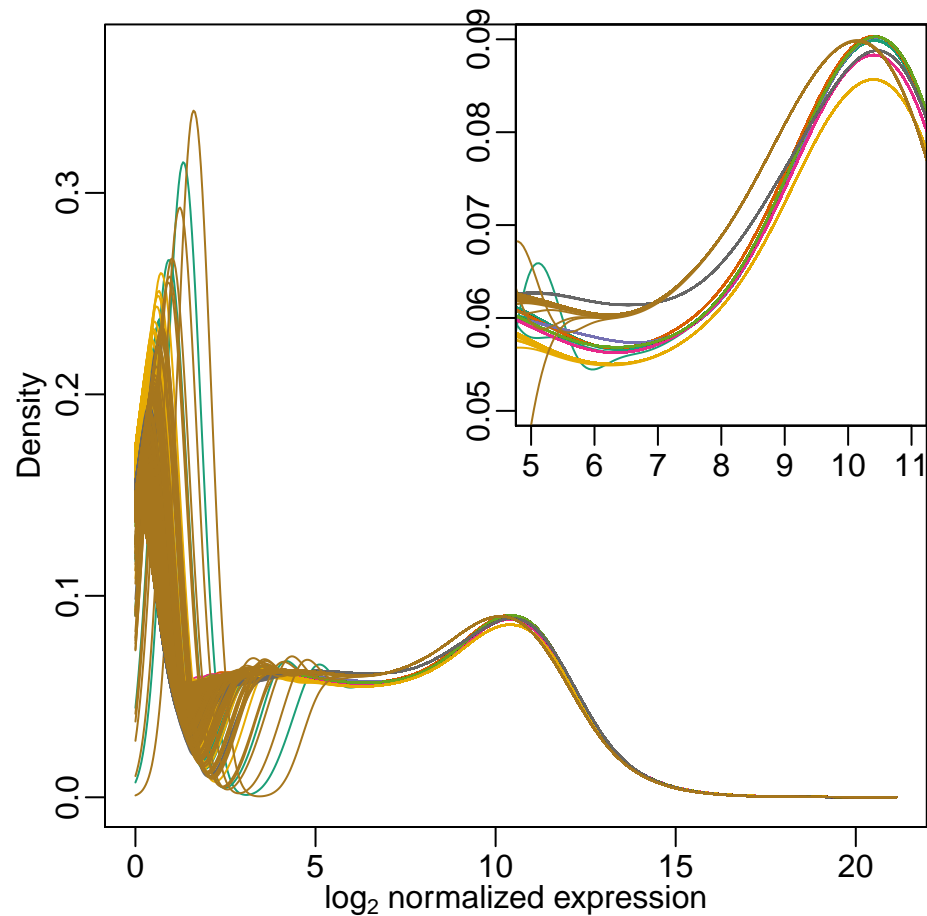

Supplement: Supplementary file 7 — Count distributions pre- and post- normalization, related to Figs. 1 and 4. Density plots of gene count distributions. Left to right: log2 raw expression distribution of samples pre-normalization; count distribution for each sample normalized in a tissue-aware manner. Colors represent different tissues. (PDF 7035 kb) [file 12859_2017_1847_MOESM7_ESM.pdf]
