# Supplementary material for: Tissue-aware RNA-Seq processing and normalization for heterogeneous and sparse data
Source: BMC Bioinformatics. 2017 Oct 3;18:437. doi: 10.1186/s12859-017-1847-x (PMC5627434; doi:10.1186/s12859-017-1847-x)
Supplement: Supplementary file 6 — Heatmap of the 15 most variable genes in the GTEx heart samples post filtering, related to Figs. 1 and 3. Heatmap of the 15 most variable genes in the GTEx heart samples. Left, top 15 genes were chosen in an unsupervised manner using the normalized gene expression after a stringent filtering in a tissue-agnostic manner. Right, the 15 most variable genes were chosen in an unsupervised manner using the normalized gene expression after tissue-specific filtering. (PDF 277 kb) [file 12859_2017_1847_MOESM6_ESM.pdf]

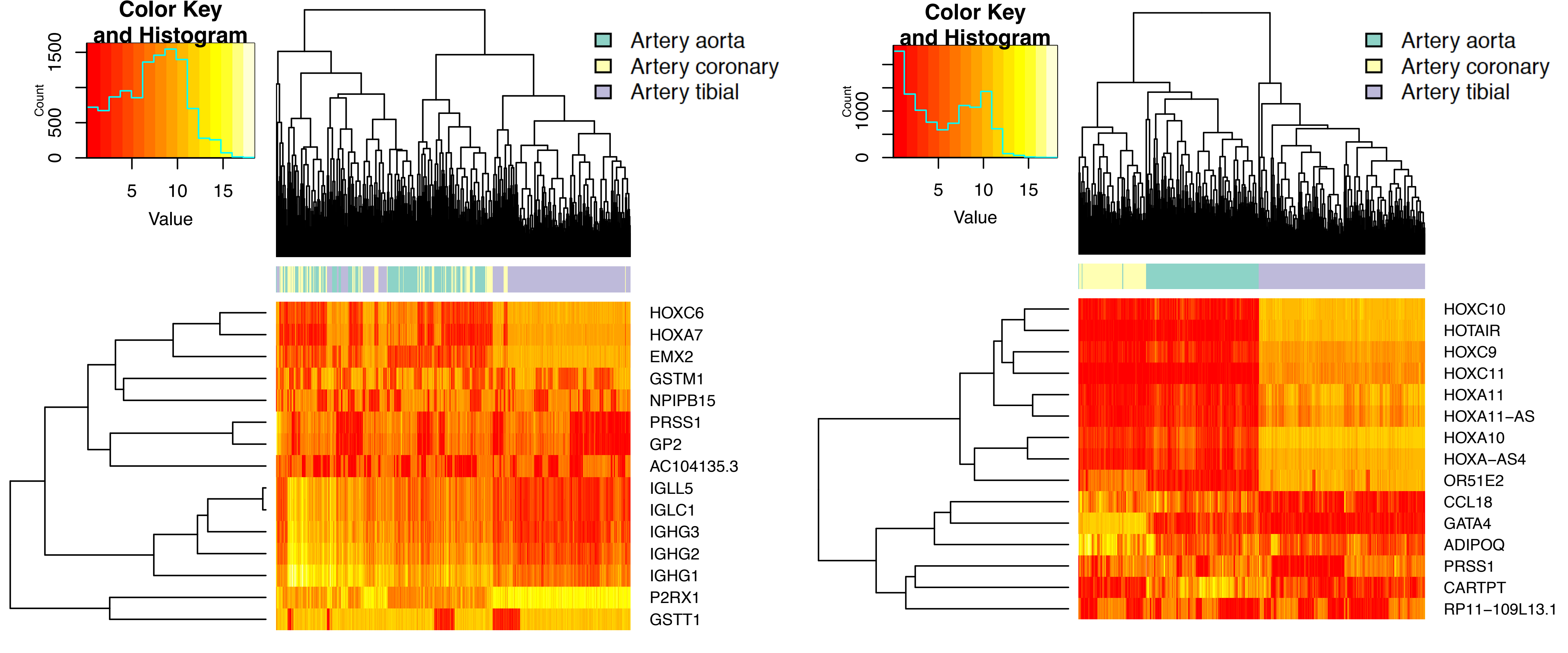
Color Key and Histogram
Count
0
500
1500
5
10
15
Value
Artery aorta
Artery coronary
Artery tibial
HOXC6
HOXA7
EMX2
GSTM1
NPIPB15
PRSS1
GP2
AC104135.3
IGLL5
IGLC1
IGHG3
IGHG2
IGHG1
P2RX1
GSTT1
Color Key and Histogram
Count
0
1000
5
10
15
Value
Artery aorta
Artery coronary
Artery tibial
HOXC10
HOTAIR
HOXC9
HOXC11
HOXA11
HOXA11−AS
HOXA10
HOXA−AS4
OR51E2
CCL18
GATA4
ADIPOQ
PRSS1
CARTPT
RP11−109L13.1
